# Dactylogyridae (Monogenoidea, Polyonchoinea) from the gills of *Auchenipterus nuchalis* (Siluriformes, Auchenipteridae) from the Tocantins River, Brazil

**DOI:** 10.1051/parasite/2020002

**Published:** 2020-01-21

**Authors:** Simone C. Cohen, Marcia C. N. Justo, Daniele V. S. Gen, Walter A. Boeger

**Affiliations:** 1 Laboratório de Helmintos Parasitos de Peixes, Instituto Oswaldo Cruz, FIOCRUZ Av. Brasil, 4365 Rio de Janeiro RJ 21045-900 Brazil; 2 Biological Interactions, Universidade Federal do Paraná Curitiba PR 81531-980 Brazil; 3 Conselho Nacional de Desenvolvimento Científico e Tecnológico, CNPq SHIS QI 01 Brasília 71.605-001 Brazil

**Keywords:** Catfish, *Cosmetocleithrum*, *Demidospermus*, Monogenea

## Abstract

Two species of *Cosmetocleithrum* Kritsky, Thatcher & Boeger, 1986 (both new) and two species of *Demidospermus* Suriano, 1983 (one new) are reported from the gills of the catfish *Auchenipterus nuchalis,* popularly known as “mapará”, from the Tocantins River and tributaries, North Region of Brazil. *Cosmetocleithrum berecae* n. sp. differs from all other species presently known in the genus by the morphology of the anchors presenting an elongate shaft and short recurved point, a coiled male copulatory organ (MCO) with three rings, and an elongate slender accessory piece with a bifurcated distal end. *Cosmetocleithrum nunani* n. sp. differs from its congeners by the combination of the following features: (1) Ventral and dorsal anchors with moderately long curved shaft and short point; (2) Hooks with poorly developed thumb; (3) Hook pairs 5 and 6 similar to each other, but morphologically distinct from remaining hook pairs; and (4) MCO coiled, with approximately 1.5 rings. *Demidospermus tocantinensis* n. sp. is easily distinguished from other species of the genus by presenting an inverted-G-shaped MCO with a median knee-like expansion. *Demidospermus osteomystax* Tavernari, Takemoto, Lacerda & Pavanelli, 2010 is redescribed based on paratypes and specimens from the gills of *A. nuchalis* from the Tocantins River, a new host and locality records for this species. The monotypic *Paracosmetocleithrum* Acosta, Scholz, Blasco-Costa, Alves & Silva, 2017, the only other Neotropical genus reported in siluriforms besides *Cosmetocleithrum* with species presenting two ribbon-like projections on the posterior margin of the dorsal bar, is considered a junior subjective synonym of *Cosmetocleithrum*.

## Abstract (fr)

Deux espèces de *Cosmetocleithrum* Kritsky, Thatcher et Boeger, 1986 (toutes deux nouvelles) et deux espèces de *Demidospermus* Suriano, 1983 (une nouvelle) sont signalées des branchies du poisson-chat *Auchenipterus nuchalis*, communément appelé « mapará », de la rivière Tocantins et affluents, Région Nord du Brésil. *Cosmetocleithrum berecae* n. sp. diffère de toutes les autres espèces actuellement connues dans le genre par la morphologie des anchors présentant un manche allongé et une pointe recourbée courte, un organe copulateur mâle (OCM) enroulé avec trois anneaux et une pièce accessoire allongée et mince avec une extrémité distale bifurquée. *Cosmetocleithrum nunani* n. sp. diffère de ses congénères par la combinaison des caractéristiques suivantes : (1) Anchors ventrales et dorsales avec tige incurvée modérément longue et pointe courte ; (2) Crochets avec pouce peu développé ; (3) Paires de crochets 5 et 6 similaires les unes aux autres, mais morphologiquement distinctes des paires de crochets restantes ; (4) OCM enroulé, avec environ 1,5 anneaux. *Demidospermus tocantinensis* n. sp. se distingue facilement des autres espèces du genre par un OCM en forme de G inversé avec une expansion médiane du genou. *Demidospermus osteomystax* Tavernari, Takemoto, Lacerda & Pavanelli, 2010 est redécrit sur la base de paratypes et de spécimens des branchies d’*A. nuchalis* de la rivière Tocantins, et représente un hôte nouveau et une localité nouvelle pour cette espèce. Le genre monotypique *Paracosmetocleithrum* Acosta, Scholz, Blasco-Costa, Alves & Silva, 2017, le seul autre genre néotropical signalé chez les Siluriformes en dehors de *Cosmetocleithrum*, avec des espèces présentant deux projections en forme de ruban sur la marge postérieure de la barre dorsale, est considéré comme synonyme subjectif junior de *Cosmetocleithrum*.

## Introduction

Siluriformes is a large and diverse order of fishes, collectively known as catfishes. Most of them are omnivores, unlike most freshwater fishes, nocturnal, and depend mainly on senses other than sight, such as tactile and chemo-sensitive barbels to explore their surroundings [20]. Siluriforms are hosts to an extraordinarily rich and diverse fauna of gill monogenoids, and this host-parasite system represents an attractive model for phylogenetic studies in the Neotropical Region [27]. The “mapará”, *Auchenipterus nuchalis* (Spix & Agassiz), inhabits lower courses of the larger rivers of South America [12]. The global fauna of the Dactylogyridae infecting catfishes is very diverse and includes around 379 species belonging to 31 genera [25]. Almost half of the genera (14) and about 75 species are native to the Neotropical Region [25]. *Demidospermus* Suriano, 1983 and *Cosmetocleithrum* Kritsky, Thatcher & Boeger, 1986 are the most diverse genera among dactylogyrids of Neotropical catfishes.

*Cosmetocleithrum* was proposed to accommodate species of dactylogyrids from *Oxydoras niger* (Valenciennes) and *Pterodoras granulosus* (Valenciennes), all from the Amazon River basin [19]. The genus includes species characterized in part by the presence of two submedial ribbon-like projections on the dorsal bar, a copulatory complex comprising a variably coiled MCO with counterclockwise rings and an elaborate non-articulated accessory piece [19]. Subsequently, several other species were proposed [1, 38]. Presently, 15 species of *Cosmetocleithrum* are known from Brazil, Argentina, and Peru [22, 27, 34, 36, 41] from siluriform hosts belonging mainly to the Doradidae but also the Auchenipteridae and Pimelodidae. A single report of a species of *Cosmetocleithrum* in *Hoplias malabaricus* (Bloch) (Erythrinidae, Characiformes) [13] requires confirmation due to the unexpected association.

*Demidospermus* was proposed to accommodate *D. anus* Suriano, 1983, a parasite from the gills of *Loricariichthys anus* (Valenciennes) [37]. Gutiérrez & Suriano [14] amended the generic diagnosis and described three new species in this genus. Subsequently, Kritsky & Gutiérrez [16] described nine more species and emended the generic diagnosis to include (1) tandem gonads (testis postgermarial); (2) a counterclockwise coiled MCO; (3) a sinistral vaginal aperture; (4) U-, W- or V-shaped haptoral bars; and (5) a sheet-like accessory piece serving as a guide for the MCO. *Demidospermus* is presently composed of 30 valid species [2, 11], all parasites of freshwater catfishes of different families, and represents one of the most species-rich Neotropical dactylogyrid genera [27]. The genus appears to be non-monophyletic based on molecular phylogenies [3, 25].

Few species of Monogenoidea were reported from fishes belonging to Auchenipteridae. These are: *Demidospermus bidiverticulatum* (Suriano & Incorvaia, 1995) and *Demidospermus osteomystax* Tavernari, Takemoto, Lacerda & Pavanelli, 2010 from *Auchenipterus osteomystax* (Miranda-Ribeiro) in Brazil; *Demidospermus centromochli* Mendoza-Franco & Scholz 2009 from *Centromochlus heckeliim* (De Filippi) in Peru; *Demidospermus uncusvalidus* Gutierrez & Suriano, 1992 from *Trachelyopterus galeatus* (L.) in Argentina; *Cosmetocleithrum striatuli* Abdallah, Azevedo & Luque, 2012 from *Trachelyopterus striatulus* (Steindachner) [9] and from *A. nuchalis* [39], *Cosmetocleithrum bulbocirrus* Kritsky, Thatcher and Boeger, 1986 from *Ageneiosus ucayalensis* [10] and *Cosmetocleithrum laciniatum* Yamada, Yamada, Silva & Anjos, 2017 from *T. galeatus* in Brazil [41]. According to Kritsky & Gutiérrez [16], the record of *D. uncusvalidus* on the auchenipterid – *T. galeatus* – by Gutiérrez & Suriano [14] is unconfirmed and may represent an undescribed species of *Demidospermus* (see [33]).

During studies on the helminth fauna of freshwater fish from Brazil, specimens of *A. nuchalis* have been examined from the Tocantins River and some tributaries in Tocantins and Maranhão, states of the North and Northeast Region of Brazil, respectively. During this study, two new species of *Cosmetocleithrum* and one new species of *Demidospermus* were described and *Demidospermus osteomystax* was redescribed from this host.

## Materials and methods

During August 2010, 67 specimens of *A. nuchalis* from the Tocantins River and two of its tributaries (Itaueiras and Arraias Rivers), and one from the Rio dos Mangues, State of Tocantins, Brazil were examined for helminths. Fishes were captured with gill nets and hook and line, the gills were removed and placed in vials containing hot water (65 °C) that were shaken; formalin was added to reach a concentration of 5%. Monogenoids were picked from the sediment and gill arches in the laboratory with the aid of a stereoscopic microscope. Some specimens were mounted in Hoyer’s medium to study the sclerotized parts and others were stained with Gomori’s trichrome to study the internal organs of the parasite [17]. Measurements are presented in micrometers; range values are followed by mean and number of structures measured in parentheses. Dimensions of organs and other structures represent the greatest distance; lengths of curved or bent structures (anchors, bars and accessory piece) represent the straight-line distances between extreme ends [19], except for *Demidospermus tocantinensis* n. sp. (see Fig. 5C). The numbering of hook pairs follows Mizelle [28] (see also [31]). Values of prevalence, mean intensity (range of intensity) and mean abundance (range of abundance) of infestation follow Bush et al. [8]. Specimens were illustrated with the aid of a camera lucida or a microprojector attached to an Olympus BX 50 microscope (both phase contrast and differential interference contrast). Type specimens were deposited in the Helminthological Collection of the Instituto Oswaldo Cruz (CHIOC) in Brazil. The holotype and paratypes of *Paracosmetocleithrum trachydorasi* Acosta, Scholz, Blasco-Costa, Alves & da Silva, 2018 (CHIOC 38.881 a–d) were examined for comparative purposes.

## Results

Systematics

Class: Monogenoidea Bychowsky, 1937

Subclass: Polyonchoinea Bychowsky, 1937

Order: Dactylogyridea Bychowsky, 1937

Dactylogyridae Bychowsky, 1933

*Cosmetocleithrum* Kritsky, Thatcher & Boeger, 1986

### *Cosmetocleithrum berecae* n. sp. (Fig. 1)

urn:lsid:zoobank.org:act:C9E53427-2373-4C26-B775-CE47881FD77F

**Figure 1:**
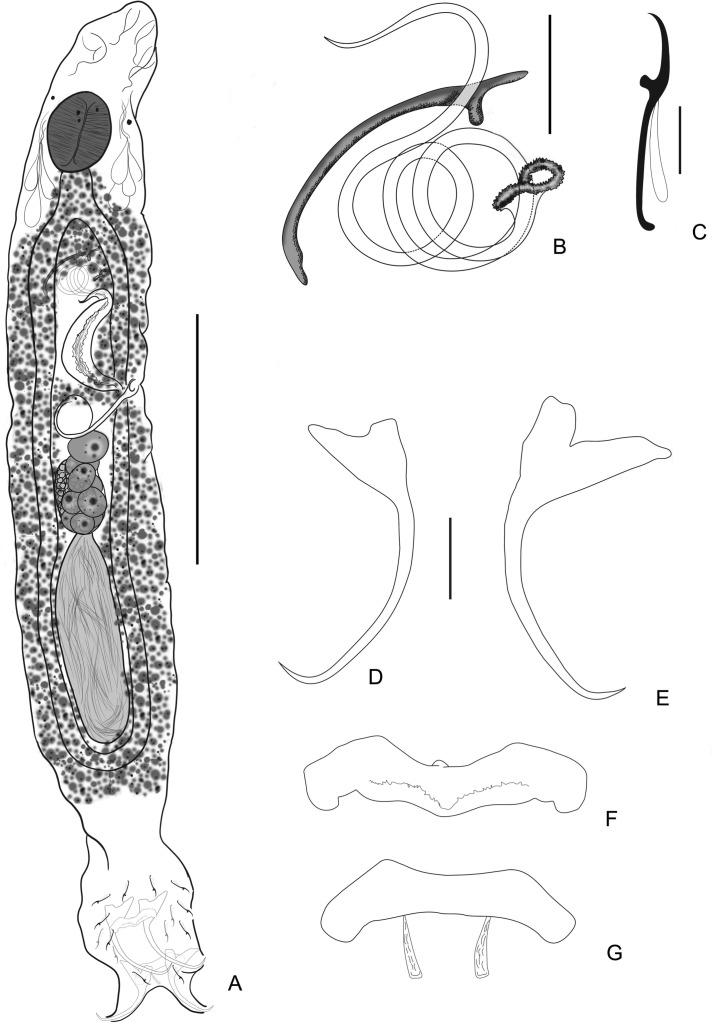
*Cosmetocleithrum berecae* n. sp. from *Auchenipterus nuchalis*. (A) Holotype, in ventral view (composite); (B) Copulatory complex, dorsal view; (C) Hook; (D) Ventral anchor; (E) Dorsal anchor; (F) Ventral bar; (G) Dorsal bar. Scale bars: (A) 100 μm; (B) 20 μm; (C) 5 μm; (D–G) 10 μm.

Type host: *Auchenipterus nuchalis* (Spix & Agassiz) (Siluriformes, Auchenipteridae).

Site: Gills.

Type-locality: Arraias River (12°37′52.3″S, 47°08′11.2″W), close to the city of Babaçulândia, State of Tocantins, Brazil.

Other localities: Tocantins River (6°32′24.53″S, 47°27′0.75″W), close to the municipality of Aguiarnópolis and Estreito; at the mouth of the Itaueiras River (6°29′58.73″S, 47°25′27.48″W), municipality of Estreito; Rio dos Mangues (10°21′40″S, 48°26′14″W), close to the municipality of Palmas, State of Tocantins, Brazil.

Infestation parameters: Total number of parasites: 276; Prevalence: 88.1% (59 hosts parasitized out of 67 examined); Mean intensity: 4.68 (1–17); Mean abundance: 4.12 (0–17).

Type-material: Holotype CHIOC 40099 a; Paratypes CHIOC 40099 b–c; 40100; 39221; 39222 a–b; 39223; 39224; 39225 a–d; 39226; 39227; 39228; 39229; 39230; 39231.

Etymology: The specific name is in honor to Dr. Berenice M. M. Fernandes, graciously called by her friends as “Bereca”, from the Instituto Oswaldo Cruz, Brazil, for her contributions to the knowledge of Brazilian helminthology.

#### Description

[Based on 42 specimens; 12 mounted in Gomori’s trichrome, 30 mounted in Hoyer’s medium]. Body fusiform, divisible into cephalic region, trunk, haptor; total body including haptor, 325–500 (410; *n* = 12) long, 72–130 (101; *n* = 8) wide. Tegument thin, smooth. Cephalic lobes moderately developed; four bilateral pairs of head organs; cephalic glands posterolateral to pharynx. Eyes not observed; accessory granules, when present, sparse in cephalic area. Mouth subterminal, midventral; pharynx 25–47 (33; *n* = 12) long, 20–45 (34; *n* = 12) wide, muscular, glandular; esophagus short and conspicuous. Two intestinal caeca confluent posteriorly to testis, lacking diverticula. Gonads in tandem (testis postgermarial), testis 40–87 (59; *n* = 8) long; vas deferens looping left intestinal caecum; seminal vesicle a dilation of vas deferens. Copulatory complex comprising male copulatory organ (MCO), accessory piece. MCO sclerotized, coiled, proximal ring 15–30 (22; *n* = 15) in diameter, with approximately three counterclockwise rings, margin of base slightly and irregularly sclerotized. Accessory piece 30–45 (35; *n* = 13) long, non-articulated with MCO, slender, with bifid distal extremity. Germarium 22–53 (40; *n* = 5) long. Oviduct, ootype, uterus not observed. Seminal receptacle globose, anterior to germarium. Vagina weakly sclerotized, tubular; vaginal aperture sinistral. Vitellaria dense throughout trunk, except in region of reproductive organs. Eggs 60–87(*n* = 2) long, 37–57 (*n* = 2) wide, ovate, without polar filaments. Haptor 77–115 (95; *n* = 8) wide, with dorsal, ventral anchor/bar complexes and 14 hooks with ancyrocephaline distribution. Ventral anchor 32–41 (36; *n* = 30) long, base 11–22 (17; *n* = 30) wide, with moderately developed tapered superficial root, short deep root, curved shaft, recurved short point, extending to the level of tip of superficial root; dorsal anchor 30–40 (34; *n* = 30) long, base 11–17 (14; *n* = 30) wide, with well-developed roots, superficial root elongate, curved shaft and straight point, extending to the level of tip of superficial root. Ventral bar 32–45 (36; *n* = 15) long, robust, broadly M-shape, with short anteromedian process; dorsal bar 30–40 (35; *n* = 10) long, robust, extremities curved posteriorly, with two submedial ribbon-like projections directed posteriorly. Hooks similar in shape, each with delicate point, protruding thumb, straight shaft, non-dilated shank, filamentous hook loop about shank length. Hook pairs equal in length, 13–20 (16; *n* = 69), except pair 5 20–22 (21; *n* = 15).

#### Remarks

There are two morphologically distinguished groups among the species of *Cosmetocleithrum*: (1) Species that resemble the type species, *Cosmetocleithrum gussevi* Kritsky, Thatcher and Boeger, 1986 that present non-articulated bars and accessory piece distally bifid, often resembling a hook (*Cosmetocleithrum parvum* Kritsky, Thatcher and Boeger, 1986, *Cosmetocleithrum rarum* Kritsky, Thatcher and Boeger, 1986, *Cosmetocleithrum sobrinus* Kritsky, Thatcher and Boeger, 1986, *Cosmetocleithrum longivaginatum* Suriano & Incorvaia, 1995, *C. striatuli*, *C. laciniatum*, *Cosmetocleithrum phryctophallus* Soares, Santos-Neto & Domingues, 2018 and *Cosmetocleithrum gigas* Morey, Cachique & Babilonia, 2019); and (2) Species depicting articulated bars with a variably shaped accessory piece (e.g. *Cosmetocleithrum confusum* Kritsky, Thatcher and Boeger, 1986, *C. bulbocirrus*, *Cosmetocleithrum tortum* Mendoza-Franco, Mendoza-Palmero & Scholz, 2016, *Cosmetocleithrum bifurcum* Mendoza-Franco, Mendoza-Palmero & Scholz, 2016).

*Cosmetocleithrum berecae* n. sp. closely resembles members of the first morphological group, differing, however, from all known species of this group by presenting anchors with an elongate shaft and a copulatory complex comprising a coiled MCO with three rings, and a slender accessory piece with well-defined and blunt elements of the bifid distal end.

### *Cosmetocleithrum nunani* n. sp. (Fig. 2)

urn:lsid:zoobank.org:act:DB1728D2-FAB7-436A-801A-39C8140200B2

**Figure 2:**
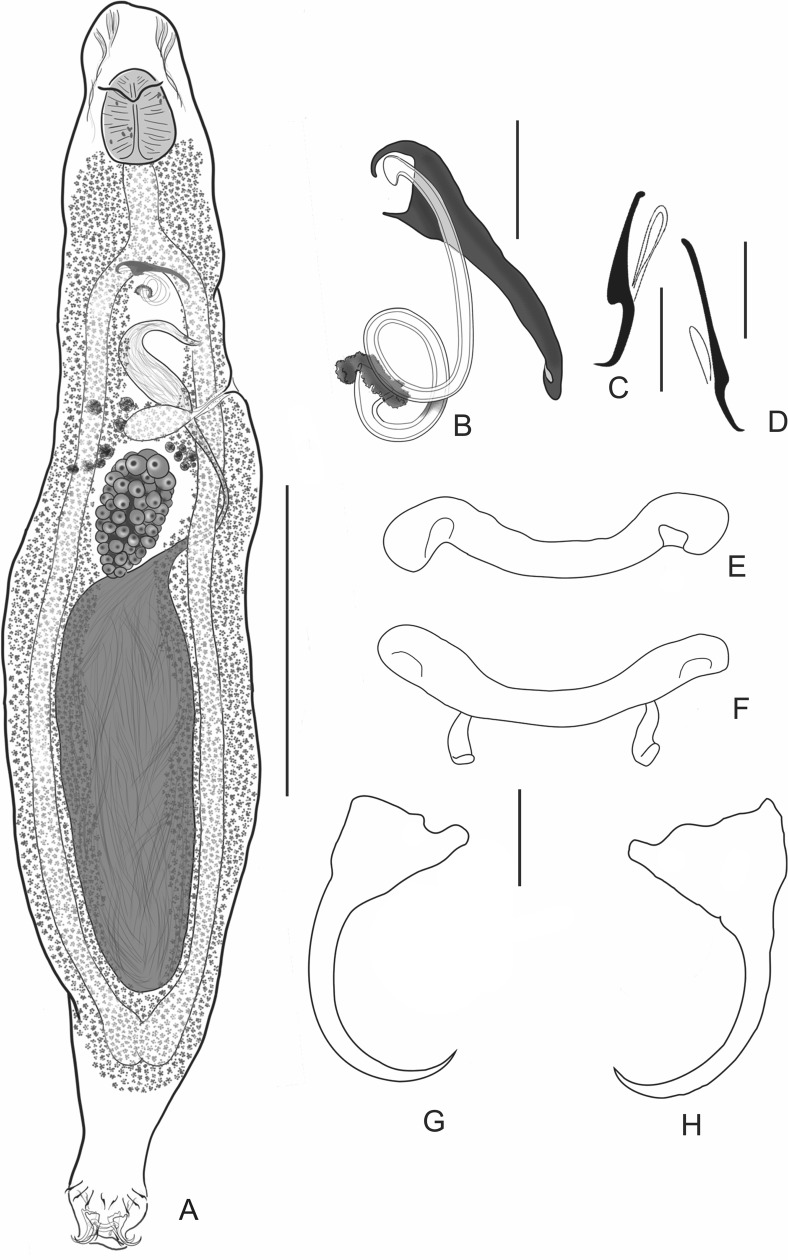
*Cosmetocleithrum nunani* n. sp. from *Auchenipterus nuchalis*. (A) Holotype, in ventral view (composite); (B) Copulatory complex, ventral view; (C) Hook pair 1; (D) Hook pair 5; (E) Ventral bar; (F) Dorsal bar; (G) Ventral anchor; (H) Dorsal anchor. Scale bars: (A) 300 μm; (B) 20 μm; (C–H) 10 μm.

Type host: *Auchenipterus nuchalis* (Spix & Agassiz) (Siluriformes, Auchenipteridae).

Type-locality: Rio dos Mangues (10°21′40″S, 48°26′14″W), close to the municipality of Palmas, State of Tocantins, Brazil.

Site: gills.

Infestation parameters: Total number of parasites: 29; Prevalence: 1.5% (1 host parasitized out of 67 examined); Intensity: 29; Mean Abundance: 0.43 (only 1 parasitized host)

Type-material: Holotype CHIOC 39232 a; Paratypes CHIOC 39232 b–z.

Etymology: The specific name is in honor to Dr. Gustavo Wilson Nunan (*in memoriam*) from the “Museu Nacional, Departamento de Vertebrados, Ictiologia, UFRJ”, Brazil, for his contribution to the knowledge of the Brazilian ichthyofauna.

#### Description

[Based on 20 specimens; 6 mounted in Gomori’s trichrome, 14 mounted in Hoyer’s medium] Body robust, divisible into cephalic region, trunk, haptor; total body including haptor 1070–1375 (1202; *n* = 7) long, 115–330 (230; *n* = 14) wide at level of germarium. Tegument smooth. Cephalic lobes poorly developed; two bilateral sets of head organs; cephalic glands indistinct. Eyes inconspicuous; accessory granules sometimes scattered in the pharyngeal region. Mouth subterminal, midventral; pharynx 80–100 (88; *n* = 5) long, 72–102 (82; *n* = 5) wide, well developed, muscular, glandular; esophagus long, well developed. Two intestinal caeca confluent just posteriorly to testis, lacking diverticula. Gonads in tandem, testis postgermarial, 170–435 (314; *n* = 9) long, 67–135 (88; *n* = 9) wide; vas deferens looping left intestinal caecum; seminal vesicle a dilatation of vas deferent. Copulatory complex comprising MCO and accessory piece. MCO sclerotized, coiled, with approximately 1.5 counterclockwise rings, widest ring 14–17 (16; *n* = 11) in diameter, with irregularly sclerotized base of MCO; Accessory piece 37–48 (42; *n* = 9) long, non-articulated, serving as a guide to MCO, rod-shaped, distally hook-shaped. Germarium 110–175 (143; *n* = 7) long, 52–125 (78; *n* = 7) wide. Mehlis’ glands immediately anterior to germarium; oviduct, uterus not observed. Seminal receptacle anterior to germarium. Vagina single, muscular; vaginal aperture sinistral. Vitellaria dense throughout trunk, except in region of reproductive organs. Eggs not observed. Peduncle conspicuous. Haptor 45–120 (94; *n* = 13) wide, subhexagonal, with dorsal, ventral anchor/bar complex and 14 hooks with ancyrocephaline distribution. Anchors similar; each with inconspicuous roots, curved shaft and point. Ventral anchor 25–30 (27; *n* = 15) long, base 15–19 (16; *n* = 5) wide; dorsal anchor 26–32 (29; *n* = 5) long, base 13–17 (15; *n* = 5) wide. Ventral bar 25–38 (35; *n* = 14) long, slightly U-shaped, ends curved posteriorly; dorsal bar 27–37 (32; *n* = 11) long slightly U-shaped with two short posteromedial ribbon-like projections. Hooks dissimilar, members of pairs 1–4, 7 more robust with recurved point, erected thumb, straight shaft, shank tapering proximally, filamentous hook (FH) loop about shank length; pairs 5–6 with delicate point, inconspicuous thumb, slightly recurved shaft, straight shank, FH loop about ⅓ shank length. Hook pairs 1–4, 7, 14–20 (17; *n* = 31); pairs 5–6, 19–22 (20; *n* = 30).

#### Remarks

*Cosmetocleithrum nunani* n. sp. closely resembles species of the first morphological group of *Cosmetocleithrum* mentioned above, which present non-articulated bars. From this group of species, the new species is most similar to *C. striatuli* and *C. laciniatum* Yamada, Yamada, Silva & Anjos, 2017. *Cosmetocleithrum nunani* n. sp can be distinguished from these species, by presenting (1) Hooks with a poorly developed thumb (conspicuous thumb in *C. striatuli* and *C. laciniatum*); (2) Hook pairs 5 and 6 morphologically distinct from remaining hooks (similar in *C. striatuli* and *C. laciniatum*); (3). Accessory piece distally hook-shaped (claw-like in *C. striatuli* and *C. laciniatum*); and (4) Body longer than 1 mm (1070–1375 μm) (*C. striatuli* varying from 564 to 898 μm and *C. laciniatum* from 295 to 617 μm).

### *Demidospermus osteomystax* Tavernari, Takemoto, Lacerda & Pavanelli, 2010 (Figs. 3 and 4)

Type host: *Auchenipterus osteomystax* (Miranda Ribeiro) (Siluriformes, Auchenipteridae).

**Figure 3:**
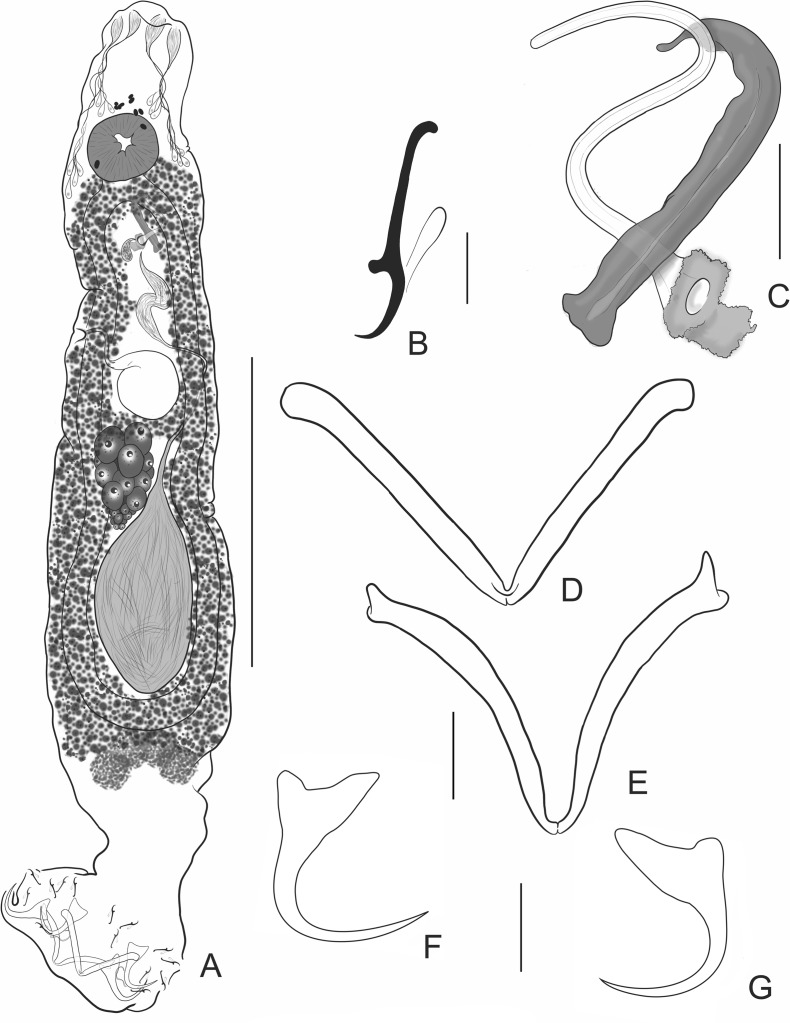
*Demidospermus osteomystax* Tavernari, Takemoto, Lacerda & Pavanelli, 2010 from *Auchenipterus nuchalis*. (A) Total, ventral view (composite); (B) Hook; (C) Copulatory complex; (D) Ventral bar; (E) Dorsal bar; (F) Ventral anchor; (G) Dorsal anchor. Scale-bars: (A) 100 μm; (B) 5 μm; (C) 10 μm; (D–G) 10 μm.

**Figure 4:**
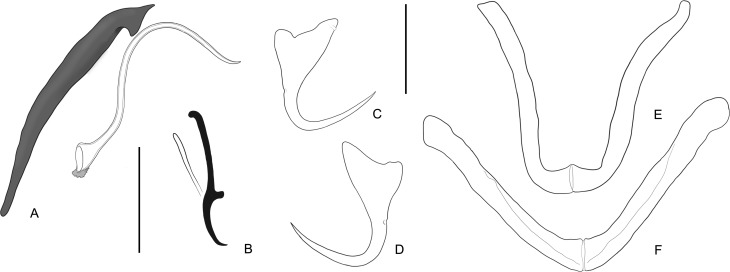
Paratypes of *Demidospermus osteomystax* Tavernari, Takemoto, Lacerda & Pavanelli, 2010 from *Auchenipterus osteomystax*. (A) Copulatory complex; (B) Hook; (C) Ventral anchor; (D) Dorsal anchor; (E) Ventral bar; (F) Dorsal bar. Scale-bars: (A, C–F) 20 μm; (B) 15 μm.

Type-locality: upper Paraná river floodplain, Brazil (22**°**50′–22**°**70′S and 53**°**15′–53**°**40′W).

Site: gills.

Current records: *Auchenipterus nuchalis* (Spix & Agassiz) (Siluriformes, Auchenipteridae from Tocantins River (6°32′24.53″S, 47°27′0.75″W), close to the municipalities of Aguiarnópolis and Estreito; at the mouth of the Itaueiras River (6°29′58.73″S, 47°25′27.48″W), municipality of Estreito, near the municipality of Babaçulândia; at the mouth of the Arraias River (12°37′52.3″S, 47°08′11.2″W), close to the municipality of Babaçulândia; Rio dos Mangues (10°21′40″S, 48°26′14″W), close to the municipality of Palmas, State of Tocantins, Brazil.

Infestation parameters: Total number of parasites: 162; Prevalence: 74.6% (50 parasitized out of 67 examined); Mean Intensity: 3.24 (1–13); Mean Abundance: 2.41 (0–13).

Specimens studied: three paratypes – CHIOC 37252-37254

Vouchers: CHIOC 39233; 39234; 39235; 39236; 39237; 39238; 39239; 39240 a–c; 39241 a–i.

#### Redescription

[Based on 30 specimens (3 paratypes and 27 vouchers from the present study), mounted in Hoyer’s medium]. Body fusiform, elongated, divisible into cephalic region, trunk and haptor. Cephalic lobes poorly developed; four pairs of head organs; cephalic glands anterolateral, posterolateral to pharynx. Eyes absent; accessory granules scattered in the cephalic region. Mouth subterminal, midventral; esophagus inconspicuous. Two intestinal caeca confluent posterior to gonads, lacking diverticula. Testis posterior, dorsal to germarium; vas deferens looping left intestinal caecum; seminal vesicle a dilation of vas deferens. Copulatory complex comprising MCO, accessory piece. MCO sclerotized, a sinuous tube, margin of base sclerotized with a conspicuous flap directed posteriorly. Accessory piece varying in shape (rod-shaped to walking-stick-shaped), non-articulated, serving as guide for MCO. Germarium pre-testicular. Oviduct, ootype, uterus not observed. Seminal receptacle anterior to germarium; globose. Vagina sclerotized; vaginal aperture dextral. Vitellaria scattered throughout the body, except for the region of reproductive organs. Eggs not observed. Haptor with dorsal, ventral anchor/bar complexes, seven pairs of hooks with ancyrocephaline distribution. Anchors similar, each with tapering superficial root, reduced deep root, short shaft, elongate point. Ventral bar articulated, strongly U/V-shaped; dorsal bar variably V-shaped, articulated medially, distal end blunt or slightly bifid. Hooks pairs similar, with short point, protruding thumb, delicate shank comprised by a single subunit.

#### Remarks

*Demidospermus osteomystax* was proposed by Tavernari et al. [40] parasitizing *Auchenipterus osteomystax* from the upper Paraná River floodplain, Brazil. The discovery of *D. osteomystax* in *A. nuchalis* represents a new host record for this species and in a new river system, the Araguaia-Tocantins basin.

The specimens collected in the Tocantins River were considered a member of this species, despite small differences (Table 1, Figs. 3 and 4) that were interpreted as intraspecific variations or artifacts of the preparation method. For instance, bars and anchors of the type specimens studied were overly flattened and may have resulted in the broad appearance of the articulation of the bars. Small differences in the morphology of the copulatory complex may represent real intraspecific features (e.g. comparatively reduced fringe around the MCO aperture in specimens of the Paraná River) (cf. Figs. 3 and 4). However, the study of specimens available from the Tocantins River and re-examination type specimens from the Paraná River clearly show that the vaginal aperture of the species is dextral, as opposed to the sinistral position described by Tavernari et al. [40].

**Table 1:** Comparative measurements (μm) of specimens of *Demidospermus osteomystax* Tavernari, Takemoto, Lacerda & Pavanelli, 2010 from different hosts and localities (*n* means number of measurements).

	Present study	*n*	Tavernari et al. (2010)	*n*
Body				
Length	190–445 (325)	10	320–540 (408)	11
Width	70–115 (87)	10	80–190 (109)	11
Haptor				
Width	73–120 (85)	14	71–108 (85)	9
Pharynx				
Length	18–23 (21)	9	25–34 (29)	5
Width	18–28 (23)	9	23–30 (26)	5
Male Copulatory Organ				
Length	10–35 (23)	10	29–48 (36)	12
Accessory piece				
Length	20–39 (25)	10	25–38 (30)	12
Ventral Bar	12–62 (32)	12	33–61 (43)	9
Dorsal Bar	18–52 (33)	12	30–50 (41)	9
Ventral Anchor	18–23 (20)	22	23–28 (25)	8
Base	8–15 (12)	22	14–16 (15)	8
Dorsal Anchor	17–23 (20)	22	21–27 (24)	8
Base	8–15 (12)	22	11–16 (15)	7
Hooks	14–17 (15)	48	13–15 (14)	10
Host	*Auchenipterus nuchalis*		*Auchenipterus osteomystax*	
Locality	Tocantins River		Upper Paraná River floodplain, Paraná, Brazil	

Due to the uncertain/questionable phylogenetic status of *Demidospermus* (as presently composed) and the absence of an emended morphological diagnosis allowing a more precise assignment of species, we tentatively retain *D. osteomystax* within this genus, while recognizing that it may represent a member of a genus-group not yet formally proposed.

### *Demidospermus tocantinensis* n. sp. (Fig. 5)

urn:lsid:zoobank.org:act:AAC27C21-D16B-4512-A759-29447089F009

**Figure 5:**
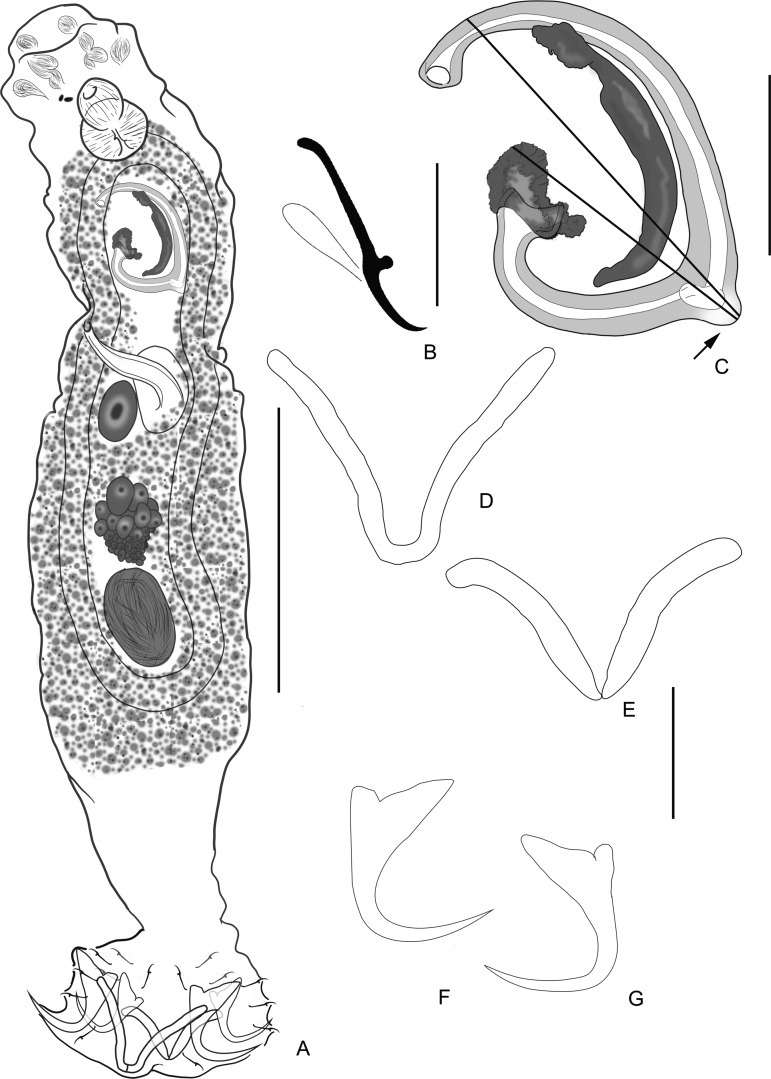
*Demidospermus tocantinensis* n. sp. from *Auchenipterus nuchalis*. (A) Total, ventral view; (B) Hook; (C) Copulatory complex, knee-like expansion of the male copulatory organ (arrow), ventral view; (D) Ventral bar; (E) Dorsal bar; (F) Ventral anchor; (G) Dorsal anchor. Scale bars: (A) 100 μm; (B) 10 μm; (C) 30 μm; (D–G) 20 μm.

Type host: *Auchenipterus nuchalis* (Spix & Agassiz) (Siluriformes, Auchenipteridae).

Type-locality: Rio dos Mangues (10°21′40″S, 48°26′14″W), close to the municipality of Palmas, State of Tocantins, Brazil.

Other localities: Tocantins River, (6°32′24.53″S, 47°27′0.75″W), close to the cities of Aguiarnópolis and Estreito; at the mouth of the Itaueiras River (6°29′58.73″S, 47°25′27.48″W), municipality of Estreito, near the city of Babaçulândia; Tocantins River, close to the mouth of the Arraias River (7°16′53.63″S, 47°41′55.05″W), State of Tocantins, Brazil.

Site: gills.

Infestation parameters: Total number of parasites: 29; Prevalence: 31.4% (21 parasitized out of 67 examined); Mean Intensity: 1.4 (1–4); Mean Abundance: 0.43 (0–4).

Type-material: Holotype CHIOC 39242 a; Paratypes CHIOC 39242 b–c; 39243; 39244; 39245; 39246; 39247; 39248.

Etymology: The specific name is derived from the type locality.

#### Description

[Based on nine specimens; two in Gomori’s trichrome, seven mounted in Hoyer’s medium]. Body fusiform, divisible into cephalic region, trunk, haptor; body (including haptor) 325–437 (378; *n* = 4) long, 55–82 (73; *n* = 4) wide at level of germarium. Cephalic margin wide; cephalic lobes poorly developed; lateral pairs of head organs; cephalic glands lateral to pharynx. Eyes absent; accessory granules scattered in cephalic region. Mouth subterminal, midventral; pharynx 25–26 (*n* = 2) in diameter; esophagus inconspicuous. Two intestinal caeca confluent posterior to gonads, lacking diverticula. Gonads in tandem; testis posterior to germarium; Testis 52–65 (*n* = 2) long, 31–35 (*n* = 2) wide; vas deferens apparently looping left intestinal caecum; seminal vesicle not observed. Copulatory complex comprising MCO, accessory piece. MCO 48–50 (50; *n* = 6) long, 29–35 (32; *n* = 6) wide, sclerotized, inverted G shaped, margin of base irregularly sclerotized, with a median knee-like expansion. Accessory piece a rod with distal irregular sclerotization, tapering proximally, non-articulated, serving as guide for MCO. Germarium 29–40 long, 15–25 (*n* = 2) wide, pre-testicular. Oviduct, ootype, uterus not observed. Seminal receptacle anterior to germarium. Vagina non-sclerotized, a wide muscular tube; vaginal aperture dextro-marginal. Vitellaria scattered throughout the body, except for the region of reproductive organs. Eggs not observed. Haptor 77–100 (90; *n* = 4) wide, with dorsal, ventral anchor/bar complexes, seven pairs of hooks with ancyrocephaline distribution. Anchors similar, each with tapering superficial root, reduced deep root, short shaft, elongate point. Ventral anchor 21–27 (24; *n* = 10) long, base 11–15 (14; *n* = 10) wide; dorsal anchor 20–25 (23; *n* = 11) long, base 12–18 (15; *n* = 11) wide. Ventral bar 38–55 (44; *n* = 5) long, U-shaped; dorsal bar 35–57 (45; *n* = 7) long, variably V-shaped, articulated medially. Hooks pairs similar, 11–17 (14; *n* = 14) long, with short point, protruding thumb, delicate shank comprised by a single subunit, filamentous hooklet (FH) loop ⅔ shank length.

#### Remarks

Like *D. osteomystax, D. tocantinensis* n. sp. also presents a dextral vagina. Similar to *D. osteomystax*, the new species is provisionally allocated to *Demidospermus* simply for the lack of more compatible generic taxon. However, similar morphology between these species (especially in the position of the vagina and the morphology of the haptoral sclerites) appear to indicate their phylogenetic proximity, which may result in subsequent proposal and assignment to another generic group. *Demidospermus centromochli*, originally assigned to *Demidospermus* and that also parasitize auchenipterids, depicts similar morphological features and may represent an additional member of this morphological (perhaps monophyletic) group. Among these species and all other species presently allocated to *Demidospermus*, *D. tocantinensis* n. sp. is easily distinguished by the unique morphology of the MCO (inverted-G shaped with a knee-like protuberance near the first third of its length).

## Discussion

It is evident from phylogenetic hypotheses (e.g. [25]) that the classification of the Dactylogyridae from freshwater Neotropical siluriforms is far from stable. This reflects the incipient knowledge about the richness and composition of species in the group. Generic groupings are being recognized continuously, representing approximately 60 years of efforts of different scientists and research groups [3, 19, 29, 30, 35, 36]. At early stages of faunal characterization, it is common to generate “catch-all” genera to accommodate morphologically similar species. This is well exemplified by the scenario revealed by Kritsky et al. [19] for the Neotropical *Urocleidoides* Mizelle and Price, 1964. Since the recognition of the morphological identity of the type species of the genus in the above-mentioned study, new generic groups have been proposed for species [15, 18, 19, 23] that could be (and were) allocated in the past to *Urocleidoides* due to the exceedingly general original diagnostic features [32].

In the present study, four species have been described or redescribed from *A. nuchalis* and allocated to two previously known genera: *Cosmetocleithrum* and *Demidospermus*. The study of the specimens from museum and freshly collected specimens of previously known species revealed further problems associated with the generic classification of the parasites of siluriforms from the Neotropics.

The fauna of monogenoids from the gills of Neotropical siluriform fishes includes species of *Demidospermus*, comprising 31 known species. According to Braga et al. [7], species of this genus display the broadest host range among siluriforms, occurring in species of Loricarioidei and Silurioidei. Other monogenoid genera with species parasitizing Neotropical freshwater siluriforms are *Cosmetocleithrum*, with eight species described from members of the Doradidae, two from Auchenipteridae and one from a pimelodid host from Brazil, Peru and Argentina; *Ameloblastella*, with 11 species from Pimelodidae, Hypophthalmidae and Heptapteridae; and *Vancleaveus*, with five species, described from species of Pimelodidae, Doradidae, and Loricariidae [4, 9, 22], the newly described *Nanayella* Acosta, Mendoza-Palmero, Silva & Scholz, 2019, with four species described from species of Pimelodidae [3], *Walteriella* Mendoza-Palmero, Mendoza-Franco, Acosta & Scholz, 2019, with two species from Pimelodidae [26], among others. Recently, *Paracosmetocleithrum* Acosta, Scholz, Blasco-Costa, Alves & Silva, 2018 was proposed for a single species, *P. trachydorasi,* from the gills of *Trachydoras paraguayensis* (Eigenmann & Ward) (Doradidae) [2].

Among the genera mentioned above, only species of two taxa are known to present two ribbon-like projections on the posterior margin of the dorsal bar: *Cosmetocleithrum* and *Paracosmetocleithrum.* The monotypic *Paracosmetocleithrum* was diagnosed by Acosta et al. [2] to include species presenting “a well-developed ornamentation in the middle portion of the ventral bar, and a sclerotized patch on the surface of the dorsal bar with an inconspicuous medial process that possesses two submedial projections arising from the tapered ends of this patch” and a dextro-marginal vagina. Acosta et al. [2] further justified the *Paracosmetocleithrum* on its position in a phylogenetic analysis based on 28S rDNA.

The morphological variability of known species of *Cosmetocleithrum* may suggest that the genus contains several subordinate clades that can be seen in the future as distinct genera following phylogenetic reconstruction of the species. Among other variable features, there are species presenting somewhat straight bars and others with V/U-shaped bars, similar to some species of *Demidospermus* (compare species proposed by Kritsky et al. [19]. However, *P. trachydorasi* is similar to the species of *Cosmetocleithrum* proposed herein and many others previously described, except for the position of the vagina. The vagina in almost all species of *Cosmetocleithrum* is reported as sinistral – except for *C. tortum* (dextral) – while the single species of *Paracosmetocleithrum* was also reported as having a dextral vagina.

A trivial comparison of the type species of *Cosmetocleithrum*, *C. gussevi,* and *P. trachydorasi* reveals their great similarity, including many of the putative features considered diagnostic for *Paracosmetocleithrum* suggested by Acosta et al. [2]. For instance, both species present similar sclerotizations on the ventral and dorsal bars, the similar general organization of the copulatory complex – with accessory piece non-articulated and distally bifurcated – egg sub-globose with a short appendix, lacking eyes, and by the presence of two ribbon-like posterior projections on the dorsal bar. Furthermore, restudy of available type specimens of *P. trachydorasi* (CHIOC 38.881 a–d) demonstrated that the original description failed in determining the ventral and dorsal orientation of the specimens studied, resulting in an error of the position of the vaginal aperture. The vaginal pore in the species is sinistral (and not dextral as originally described). For the same reason, the illustration mistakenly depicts the vas deferens looping the right caecum. While both species appear unique, they are clearly congeneric. Since *Cosmetocleithrum* has priority over *Paracosmetocleithrum*, *P. trachydorasi* is transferred to *Cosmetocleithrum* as *C. trachydorasi* comb. nov. *Paracosmetocleithrum* is a junior subjective synonym of *Cosmetocleithrum*.

Additionally, the phylogenetic independence of *Paracosmetocleithrum* suggested by the hypothesis of Acosta et al. [2] is questionable. The reduced support for its sister-group relationships and the impossibility of testing its monophyly are deceptive. By having a single species, *Paracosmetocleithrum* is monophyletic by assumption. Also, the low values of posterior probability and/or bootstrap of several putative ancestral branches of the phylogeny used to justify the identity of *Paracosmetocleithrum* (see Figure 3 of Acosta et al. [2]) suggest a soft polytomy (see [21]) which includes *P. trachydorasi*, two species of *Cosmetocleithrum,* and two clades including species of *Demidospermus* and unidentified dactylogyrids.

As suggested by Franceschini et al. [11], *Demidospermus* likely represents an unnatural taxon due to the recurrent polyphyletic nature of its current members in several phylogenetic hypotheses based on molecular data [11, 25, 26]. Unfortunately, previous phylogenetic studies did not explore the evolution of morphological features, which could provide support for a temporary or a definitive resolution to the problem. The integration of molecular and morphological data into a more comprehensive phylogenetic hypothesis can potentially provide diagnostic features for generic groups, as demonstrated by Mendoza-Palmero et al. [26]. However, even in the absence of molecular data, a comprehensive analysis of morphological features can provide important evidence for yet undisclosed supraspecific groupings within the apparently polyphyletic *Demidospermus.* The critical analysis of museum and freshly collected specimens, and descriptions of the known species reveals distinctive features (synapomorphies?) of dactylogyrids of auchenipterid hosts presently allocated to *Demidospermus*.

For instance, all five species of *Demidospermus* from auchenipterid hosts have a morphology similar to other species of *Demidospermus,* except for two species, *D. osteomystax* and *D. tocantinensis,* both of which present a dextral vagina. Examination of the whole mount illustrated in the original description of *D. centromochli* (Fig. 1 of [24]) suggests that the vagina may also be dextral, based on the pathway of the vas deferens that invariably loops the left caecum in Dactylogyridae [5, 6]. Since the report of *D. uncusvalidus* on an Auchenipteridae is likely erroneous [16], the only species presently allocated to *Demidospermus* from this family of hosts that depicts sinistral vagina is *D. bidiverticulatum*. Sharing of a dextral vagina and similarities on the morphology of the MCO appears to suggest common ancestry of these species and may result in the proposal of a new genus.

However, to avoid additional instability to the already confused classification of dactylogyrids of freshwater Neotropical siluriforms, the proposal of a new genus is not considered desirable at this moment. For this reason, *D. osteomystax* and *D. tocantinensis* are provisionally retained in *Demidospermus* until both morphological data and molecular markers are available for them and several representatives of present and future known species from these hosts, which may result in the recognition of monophyletic groups and their respective morphological synapomorphies that will allow the establishment of a more robust classification of the group. While the use of molecular markers may generate information important to the reorganization of the classification of these dactylogyrids (and any other group), this should not signify that morphological analyses and interpretations can be ignored or belittled. Unfortunately, this seems to be the general pattern of many recent publications on Neotropical Monogenoidea. The published errors in determination of the dorsoventral axis of mounted specimens resulting in the wrong allocation of the vagina and other sclerotized structures in recent taxonomic accounts of the group and other serious mistakes in morphological interpretations, are consistent with this.

## References

[1] Abdallah VD, Azevedo RK, Luque JL. 2012 Three new species of Monogenea (Platyhelminthes) parasites of fish in the Guandu River, southeastern Brazil. Acta Scientiarium Animal Science, 34, 483–490.

[2] Acosta AA, Scholz T, Blasco-Costa I, Alves PV, Silva RJ. 2018 A new genus and two new species of dactylogyrid monogeneans from gills of Neotropical catfishes (Siluriformes: Doradidae and Loricariidae). Parasitology International, 67, 4–12. 28939534 10.1016/j.parint.2017.09.012

[3] Acosta AA, Mendoza-Palmero CA, Silva RJ, Scholz T. 2019 A new genus and four new species of dactylogyrids (Monogenea), gill parasites of pimelodid catfishes (Siluriformes: Pimelodidae) in South America and the reassignment of *Urocleidoides megorchis* Mizelle et Kritsky, 1969. Folia Parasitologica, 66, 004. 10.14411/fp.2019.004 30964045

[4] Boeger WA, Cohen SC, Domingues MV, Justo MCN, Pariselle A. 2018 Monogenoidea. Catálogo Taxonômico da Fauna do Brasil. PNUD Available from http://fauna.jbrj.gov.br/fauna/faunadobrasil/2719. Acessed on 16 August 2018.

[5] Boeger WA, Kritsky DC. 1997 Coevolution of the Monogenoidea (Platyhelminthes) based on a revised hypothesis of a parasite phylogeny. International Journal for Parasitology, 27, 1495–1511. 9467734 10.1016/s0020-7519(97)00140-9

[6] Boeger WA, Kritsky DC. 2001 Interrelationships of the Monogenoidea, in Interrelashionships of Platyhelminthes, Littlewood TTJ, Bray RA, Editors. Taylor & Francis, New York p. 92–102.

[7] Braga MP, Araujo SB, Boeger WA. 2014 Patterns of interaction between Neotropical freshwater fishes and their gill Monogenoidea (Platyhelminthes). Parasitology Research, 113, 481–490. 24221891 10.1007/s00436-013-3677-8

[8] Bush AO, Lafferty KD, Lotz JM, Shostak W. 1997 Parasitology meets ecology on its own terms: Margolis et al., revisited. Journal of Parasitology, 83, 575–583. 9267395

[9] Cohen SC, Justo MCN, Kohn A. 2013 South American Monogenoidea parasites of fishes, amphibians and reptiles, 1st edn Rio de Janeiro: Oficina de livros p. 663.

[10] Ferreira DO, Tavares-Dias M. 2017 Ectoparasites and endoparasites community of *Ageneiosus ucayalensis* (Siluriformes: Auchenipteridae), catfish from Amazon River system in northern Brazil. Journal of Parasitic Diseases, 41, 639–646. 28848252 10.1007/s12639-016-0857-3 PMC5555905

[11] Franceschini L, Zago AZ, Müller MI, Francisco CJ, Takemoto RM, Silva RJ. 2018 Morphology and molecular characterization of *Demidospermus spirophallus* n. sp., *D. prolixus* n. sp. (Monogenea: Dactylogyridae) and a redescription of *D. anus* in siluriform catfish from Brazil. Journal of Helminthology, 92, 228–243. 28382887 10.1017/S0022149X17000256

[12] Froese R, Pauly D, Editors. 2019 FishBase. World Wide Web electronic publication. www.fishbase.org. Version (04/2019).

[13] Graça RJ, Ueda BH, Oda FH, Takemoto RM. 2013 Monogenea (Platyhelminthes) parasites from the gills of *Hoplias* aff. *malabaricus* (Bloch, 1794) (Pisces: Erythrinidae) in the Upper Paraná River Floodplain, States of Paraná and Mato Grosso do Sul, Brazil. Check List, 9, 1484–1487.

[14] Gutiérrez PA, Suriano DM. 1992 Ancyrocephalids of the genus *Demidospermus* Suriano, 1983 (Monogenea) parasites from Siluriform fishes in Argentina, with descriptions of three new species. Acta Parasitologica, 37, 169–172.

[15] Jogunoori W, Kritsky DC, Venkatanarasaiah J. 2004 Neotropical Monogenoidea. 46. Three new species from the gills of introduced aquarium fishes in India, the proposal of *Heterotylus* n. g. and *Diaphorocleidus* n. g., and the reassignment of some previously described species of *Urocleidoides* Mizelle & Price, 1964 (Polyonchoinea: Dactylogyridae). Systematic Parasitology, 58, 115–124. 15449827 10.1023/b:sypa.0000029422.16712.9a

[16] Kristky DC, Gutiérrez PA. 1998 Neotropical Monogenoidea. 34. Species of *Demidospermus* (Dactylogyridae, Ancyrocephalinae) from the gills of pimelodids (Teleostei, Siluriformes) in Argentina. Journal of the Helminthological Society of Washington, 65, 147–159.

[17] Kritsky DC, Leiby PD, Kayton RJ. 1978 A rapid stain technique for the haptoral bars of *Gyrodactylus* species (Monogenea). Journal of Parasitology, 64, 172–174. 76672

[18] Kritsky DC, Mendoza-Franco EF, Scholz T. 2000 Neotropical Monogenoidea. 36. Dactylogyrids from the gills of *Rhamdia guatemalensis* (Gunther) (Siluriformes: Pimelodidae) from cenotes of the Yucatan Peninsula, Mexico, with proposal of *Ameloblastella* gen. n. and *Aphanoblastella* gen. n. (Dactylogyridae: Ancyrocephalinae). Comparative Parasitology, 67, 76–84.

[19] Kristky DC, Thatcher VE, Boeger WA. 1986 Neotropical Monogenea. 8. Revision of *Urocleidoides* (Dactylogyridae, Ancyrocephalinae). Proceedings of the Helminthological Society of Washington, 53, 1–37.

[20] Lundberg JG, Friel JP. 2003 Siluriformes. Catfishes, version 20 January 2003 (under construction). http://tolweb.org/Siluroformes/15065/2003.01.20 *in* The Tree of Life Web Project (http://tolweb.org).

[21] Maddison W. 1989 Reconstructing character evolution on polytomous cladograms. Cladistics, 5, 365–377. 10.1111/j.1096-0031.1989.tb00569.x 34933477

[22] Mendoza-Franco EF, Mendoza-Palmero CA, Scholz T. 2016 New species of *Ameloblastella* Kritsky, Mendoza-Franco & Scholz, 2000 and *Cosmetocleithrum* Kritsky, Thatcher & Boeger, 1986 (Monogenea: Dactylogyridae) infecting the gills of catfishes (Siluriformes) from the Peruvian Amazonia. Systematic Parasitology, 93, 847–862. 27743238 10.1007/s11230-016-9671-7

[23] Mendoza-Franco EF, Reina RG, Torchin ME. 2009 Dactylogyrids (Monogenoidea) parasitizing the gills of *Astyanax* spp. (Characidae) from Panama and Southeast Mexico, a new species of *Diaphorocleidus* and a proposal for *Characithecium* n. gen. Journal of Parasitology, 95, 46–55. 19245277 10.1645/GE-1592.1

[24] Mendoza-Franco EF, Scholz T. 2009 New Dactylogyrids (Monogenea) Parasitizing the gills of catfishes (Siluriformes) from the Amazon River Basin in Peru. Journal of Parasitology, 95, 865–870. 19215149 10.1645/GE-1820.1

[25] Mendoza-Palmero CA, Blasco-Costa I, Scholz T. 2015 Molecular phylogeny of Neotropical monogeneans (Platyhelminthes: Monogenea) from catfishes (Siluriformes). Parasites & Vectors, 8, 164. 25890068 10.1186/s13071-015-0767-8 PMC4374382

[26] Mendoza-Palmero CA, Mendoza-Franco E, Acosta A, Scholz T. 2019 *Walteriella* n. g. (Monogenoidea: Dactylogyridae) from the gills of pimelodid catfishes (Siluriformes: Pimelodidae) from the Peruvian Amazonia based on morphological and molecular data. Systematic Parasitology, 96, 441–452. 31165371 10.1007/s11230-019-09866-8

[27] Mendoza-Palmero CA, Scholz T, Mendoza-Franco EF, Kuchta R. 2012 New species and geographical records of dactylogyrids (Monogenea) of Catfish (Siluriformes) from the peruvian Amazonia. Journal of Parasitology, 98, 484–497. 22191552 10.1645/GE-2941.1

[28] Mizelle JD. 1936 New species of trematodes from the gills of Illinois fishes. American Midland Naturalist, 17, 785–806.

[29] Mizelle JD, Kritsky DC. 1969 Studies on monogenetic trematodes. XXXIX. Exotic species of Monopisthocotylea with the proposal of *Archidiplectanum* gen. n. and *Longihaptor* gen. n. American Midland Naturalist, 81, 370–386.

[30] Mizelle JD, Kritsky DC, Crane JW. 1968 Studies on monogenetic trematodes. XXXVIII. Ancyrocephalinae from South America with the proposal of *Jainus* gen. n. American Midland Naturalist, 80, 186–198.

[31] Mizelle JD, Price CE. 1963 Additional haptoral hooks in the genus *Dactylogyrus*. Journal of Parasitology, 49, 1028–1029.

[32] Mizelle JD, Price CE. 1964 Studies on monogenetic trematodes. XXVII. Dactylogyrid species with the proposal of *Urocleidoides* gen. n. Journal of Parasitology, 50, 579–584. 14206483

[33] Monteiro CM, Kritsky DC, Brasil-Sato MC. 2010 Neotropical Monogenoidea. 55. Dactylogyrids parasitizing the pintado-amarelo *Pimelodus maculatus* Lacepede (Actinopterygii: Pimelodidae) from the Rio Sao Francisco. Brazil. Systematic Parasitology, 76, 179–190. 20532849 10.1007/s11230-010-9250-2

[34] Morey GAM, Cachique JCZ, Babilonia JJS. 2019 *Cosmetocleithrum gigas* sp. n. (Monogenoidea: Dactylogyridae) from the gills of *Oxidoras niger* (Siluriformes: Doradidae) from the Peruvian Amazon. Biologia 74, 1–4.

[35] Negreiros LP, Tavares-Dias M, Pereira FB. 2019 Monogeneans of the catfish *Pimelodus blochii* Valenciennes (Siluriformes: Pimelodidae) from the Brazilian Amazon, with a description of a new species of *Ameloblastella* Kritsky, Mendoza-Franco & Scholz, 2000 (Monogenea: Dactylogyridae). Systematic Parasitology, 96, 399–407. 31087201 10.1007/s11230-019-09862-y

[36] Soares GB, Neto JFS, Domingues MV. 2018 Dactylogyrids (Platyhelminthes: Monogenoidea) from the gills of *Hassar gabiru* and *Hassar orestis* (Siluriformes: Doradidae) from the Xingu Basin, Brazil. Zoologia, 35, e23917.

[37] Suriano DM. 1983 *Demidospermus anus* gen. nov. sp. nov. (Monogenea, Ancyrocephalinae) parasita branquial de *Loricaria (L*.) *anus* Valenciennes, 1840 (Pisces: Loricariidae) de la Laguna de Chascomus, Provincia de Buenos Aires, Republica Argentina. Neotropica, 29, 111–119.

[38] Suriano DM, Incorvaia IS. 1995 Ancyrocephalid (Monogenea) parasites from siluriform fishes from the Paranean Platean icthyogeographical province in Argentine. Acta Parasitologica, 40, 113–124.

[39] Tavares-Dias M. 2017 Community of protozoans and metazoans parasitizing *Auchenipterus nuchalis* (Auchenipteridae), a catfish from the Brazilian Amazon. Acta Scientiarium Biological Sciences, 39, 123–128.

[40] Tavernari FC, Takemoto RM, Lacerda ACF, Pavanelli GC. 2010 A new species of *Demidospermus* Suriano, 1983 (Monogenea) parasite of gills of *Auchenipterus osteomystax* (Auchenipteridae), from the upper Parana river floodplain, Brazil. Acta Scientiarium Biological Sciences, 32, 79–81.

[41] Yamada POF, Yamada FH, Silva RJ, Anjos LA. 2017 A new species of *Cosmetocleithrum* (Monogenea, Dactylogyridae), a gill parasite of *Trachelyopterus galeatus* (Siluriformes, Auchenipteridae) from Brazil, with notes on the morphology of *Cosmetocleithrum striatuli*. Comparative Parasitology, 84, 119–123.

